# Premenopausal Singaporean Women Suffering from Major Depressive Disorder Treated with Selective Serotonin Reuptake Inhibitors Had Similar Bone Mineral Density as Compared with Healthy Controls

**DOI:** 10.3390/diagnostics12010096

**Published:** 2022-01-03

**Authors:** Roger C. Ho, Anna N. Chua, Syeda Fabeha Husain, Wanqiu Tan, Fengyi Hao, Giang T. Vu, Bach X. Tran, Hien Thu Nguyen, Roger S. McIntyre, Cyrus S. Ho

**Affiliations:** 1 Department of Psychological Medicine, Yong Loo Lin School of Medicine, National University of Singapore, Singapore 119228, Singapore; pcmrhcm@nus.edu.sg (R.C.H.); annacyz@gmail.com (A.N.C.); pcmhsh@nus.edu.sg (C.S.H.); 2 Institute for Health Innovation and Technology (iHealthtech), National University of Singapore, Singapore 119077, Singapore; 3 Department of Paediatrics, Yong Loo Lin School of Medicine, National University of Singapore, Singapore 119228, Singapore; 4 Research Institute, National University of Singapore (Chongqing), Chongqing 401123, China; wanqiu.tan@nusricq.cn; 5 Sleep Medicine Center, Department of Respiratory and Critical Care Medicine, Mental Health Center, Translational Neuroscience Center, and State Key Laboratory of Biotherapy, West China Hospital, Sichuan University, Dian Xin Nan Jie 28, Chengdu 610041, China; fengyihao@cqljrmyy.com; 6 Center of Excellence in Behavioural Medicine, Nguyen Tat Thanh University, Ho Chi Minh City 60000, Vietnam; giang.coentt@gmail.com; 7 Institute for Preventive Medicine and Public Health, Hanoi Medical University, Hanoi 70000, Vietnam; bach@jhu.edu; 8 Bloomberg School of Public Health, Johns Hopkins University, Baltimore, MD 21205, USA; 9 Institute for Global Health Innovations, Duy Tan University, Da Nang 550000, Vietnam; hien.nguyen.ighi@gmail.com; 10 Mood Disorders Psychopharmacology Unit, University Health Network, University of Toronto, Toronto, ON M5G 2C4, Canada; roger.mcintyre@uhn.ca

**Keywords:** bone mineral density, major depressive disorder, premenopause, selective serotonin reuptake inhibitor (SSRI), women

## Abstract

The association between selective serotonin reuptake inhibitor (SSRI) treatment and lower bone mineral density (BMD) remains controversial, and further research is required. This study aimed to compare the BMD, levels of bone formation and bone metabolism markers in medicated premenopausal Singaporean women with major depressive disorder (MDD) and matched healthy controls. We examined 45 women with MDD who received SSRI treatment (mean age: 37.64 ± 7) and 45 healthy controls (mean age: 38.1 ± 9.2). BMD at the lumbar spine, total hip and femoral neck were measured using dual-energy X-ray absorptiometry. We also measured bone formation markers, procollagen type 1 N-terminal propeptide (P1NP) and bone metabolism markers, osteoprotegerin (OPG) and receptor activator of nuclear factor-kappa-Β ligand (RANKL). There were no significant differences in the mean BMD in the lumbar spine (healthy controls: 1.04 ± 0.173 vs. MDD patients: 1.024 ± 0.145, *p* = 0.617, left hip (healthy controls: 0.823 ± 0.117 vs. MDD patients: 0.861 ± 0.146, *p* = 0.181) and right hip (healthy controls: 0.843 ± 0.117 vs. MDD patients: 0.85 ± 0.135, *p* = 0.784) between healthy controls and medicated patients with MDD. There were no significant differences in median P1NP (healthy controls: 35.9 vs. MDD patients: 37.3, *p* = 0.635), OPG (healthy controls: 2.6 vs. MDD patients: 2.7, *p* = 0.545), RANKL (healthy controls: 23.4 vs. MDD patients: 2178.93, *p* = 0.279) and RANKL/OPG ratio (healthy controls: 4.1 vs. MDD patients: 741.4, *p* = 0.279) between healthy controls and medicated patients with MDD. Chronic SSRI treatment might not be associated with low BMD in premenopausal Singaporean women who suffered from MDD. This finding may help female patients with MDD make an informed decision when considering the risks and benefits of SSRI treatment.

## 1. Introduction

Major depressive disorder (MDD) is a common psychiatric disorder with a lifetime prevalence of 10.8% [1]. Antidepressant medication represents a treatment option in people suffering from MDD. Selective serotonin reuptake inhibitors (SSRI), including fluoxetine, paroxetine and sertraline, have the highest popularity index among all biological treatments for MDD [2]. A previous study found that premenopausal women suffering from MDD had low bone mineral density (BMD) and high bone metabolism turnover [3]. Another study reported that patients treated with antidepressants might have decreased BMD, putting the patients at an increased risk of fractures [4]. Consequently, it has been reported that patients receiving antidepressant treatment may be at increased risk for osteoporosis or osteoporotic fractures [5].

Notwithstanding, other studies have failed to find an association between SSRI prescription and increased rate of bone loss. Diem et al. found no evidence that SSRI had an increased rate of bone loss in American users compared to non-users [6]. Herrou et al. did not find an association between SSRI treatment and a low BMD in a sample in France [7]. In the current literature, no study performed on multi-ethnic Singaporeans evaluating SSRIs has been associated with low BMD.

There are several research gaps that need to be addressed. First, MDD patients from different ethnic backgrounds might have different views on treatment [8], behavior and lifestyles. Race and ethnicity are important determinants of risk for osteopenia and osteoporosis [9]. Studies that reported the association between SSRI use and low BMD were conducted in Europeans [3] and North Americans [4]. These findings might not be generalizable to other ethnicities. Further study is required to investigate the relationship between SSRI use and BMD in Singaporeans. Second, the relationship between SSRI use and levels of bone formation markers, including procollagen type 1 N-terminal propeptide (P1NP) [10], as well as bone metabolism markers, such as the receptor activator of nuclearfactor-kappa-Β ligand (RANKL) and osteoprotegerin (OPG), were conducted in animal studies [11,12,13]. These findings may not be translatable to patients in a clinical setting. The formation of type 1 collagen represents bone formation and osteoblast function because procollagen is cleaved at the C and N terminals during bone formation. Hence, the levels of P1NP reflect the rate of new bone formation [14]. RANKL activates osteoclast formation and increases bone turnover [15]. In contrast, OPG protects bone from excessive resorption by binding to RANKL and preventing it from binding to the receptor activator of nuclearfactor-kappa-Β (RANK) [15]. The RANKL/OPG ratio is an important determinant of bone mass and skeletal integrity. A further clinical study is required to compare the levels of bone formation (i.e., P1NP) and metabolism markers between patients who receive SSRI treatment and healthy controls who are unmedicated. Third, non-adherence to SSRIs among people with MDD is a public health concern because it led to poor functioning at work [16], high indirect costs due to loss of functioning [17] and potential complications such as suicide [18]. A common misconception about SSRIs includes the association between SSRI use and suicidal ideation [19] and potential addiction. Recently, a study highlighted that misconceptions or negative beliefs about SSRIs could lead to poor adherence [20]. The belief about SSRIs causing osteopenia or osteoporosis would have a negative impact on adherence and increases risk of relapse or poor control of depressive symptoms. As a result, further research is needed to study the relationship between antidepressant SSRI use, bone markers and BMD, with careful selection of patients with MDD and controls to minimize potential confounding factors.

In order to determine whether SSRI use among Singaporean premenopausal women is associated with low BMD, this study aimed to compare the BMD, levels of bone formation and bone metabolism markers in medicated premenopausal women with MDD and healthy controls. This study has two hypotheses: (1) premenopausal women who suffer from MDD and are being treated with SSRI treatment exhibit lower BMD when compared to healthy controls; (2) premenopausal women who are diagnosed with MDD and are being treated with SSRI treatment exhibit evidence of lower bone formation (i.e., P1NP) but higher bone metabolism markers (i.e., RANKL, OPG and RANKL/OPG ratio).

## 2. Materials and Methods

### 2.1. Study Design and Participants

This project was a cross-sectional study comparing BMD between premenopausal women suffering from MDD, and healthy controls. Patients and healthy controls were recruited from the National University Hospital Depressive Disorder Clinic and the community in Singapore between 1 April 2008 and 26 February 2013. During the recruitment period, the patients were recruited first, and controls were recruited subsequently. These female controls were matched with patients based on age, ethnicities, BMI, menstruation parameters and smoking status, because these confounding factors could affect BMD. Inclusion criteria for patients were as follows: (i) female gender; (ii) aged between 21 and 50 years; (iii) satisfying the Diagnostic and Statistical Manual (DSM)–IV criteria for MDD; (iv) treated with antidepressants; and (v) premenopausal. Healthy controls were recruited from the community through word of mouth, and potential participants were assessed based on inclusion and exclusion criteria. The healthy controls underwent the same scans and venepuncture procedures as the patients with MDD. All participants had two ovaries, an intact uterus and were not on estrogen or other medications that affected ovarian function (for example, oral contraceptive pills). The following were the exclusion criteria for patients and controls: (1) presence of alcohol, tobacco and substance use conditions (for example, cannabis, amphetamine); (2) regular usage of calcium and vitamin D supplements; (3) presence of chronic medical conditions (for example, myocardial infarction, cancer); (4) pregnancy or menopause; (5) lack of mental capacity to give consent; and (6) presence of other psychiatric disorders (for example, schizophrenia, bipolar disorder). The sample size was based on a previous study on BMD and psychiatric patients with a sample size from 32 to 70 per diagnostic group [7]. We originally recruited 58 medicated patients with MDD. Based on the reviewer’s recommendation, this study should focus on the most commonly prescribed antidepressant among the participants, SSRI. We excluded 13 patients from the analysis because they had been prescribed other types of antidepressants, including serotonin–norepinephrine reuptake inhibitor (SNRI), noradrenergic and specific serotonergic antidepressant (NaSSa) and tricyclic antidepressant (TCA).

### 2.2. Measures and Instruments

The severity of depression was assessed using the 21-item Hamilton Depression Rating Scale (HAM-D) previously validated in Singaporeans [17]. The Ham-D is a commonly used depression assessment scale administered by psychiatrists or trained research assistants. The HAM-D covers the following depressive symptom in the past week: depressed mood, feelings of guilt, suicide, initial, middle and terminal insomnia, work and interests, psychomotor retardation, agitation, psychiatric anxiety, somatic anxiety, gastrointestinal somatic symptoms, genital symptoms, hypochondriasis, weight loss, insight, diurnal variation of mood, depersonalization and derealization, paranoid symptoms and compulsive behavior [21]. Disease severity based on HAMD scores is 0–10: mild depression; 11–23: moderate depression; >23: severe depression [22].

Sera were obtained by centrifuging blood collected by venipuncture during routine laboratory tests. Cholesterol (within-day assay CV 0.53% to 1.45% for a concentration range of 2.60 to 7.25 mmol/L, between-day assay CV 1.00% to 1.33% for a concentration range of 2.60 to 7.25 mmol/L), high-density lipoprotein (HDL) (within-day assay CV 0.56% to 0.65% for a concentration range of 0.9 to 2.2 mmol/L, between-day assay CV 1.18% to 3.53% for a concentration range of 0.9 to 2.2 mmol/L), and triglycerides (within-day assay CV 0.00 to 3.85% for a concentration range of 1.6 to 5.1 mmol/L, between-day assay CV 1.24% to 3.40% for a concentration range of 1.6 to 5.1 mmol/L) were measured using a Siemens Advia 2400 (Siemens, Munich, Germany). Cholesterol was measured using the enzymatic method, HDL using the elimination/catalase method and triglycerides using GPO, Trinder without serum blank.

OPG and RANKL concentrations were determined in serum using a highly sensitive, commercial ELISA provided by Immundiagnostik (Bensheim, Germany). Measurements were performed in samples according to manufacturer’s instructions. The lower limit of detection of this assay is 2.8 pg/mL; the intraassay and interassay (*n* = 16) coefficient of variation is <10%. All assays were measured blinded to any clinical information and performed in duplicate. The lower limit of detection of this assay is 2.8 pg/mL for OPG and 8 pg/mL for RANK-L. The intraassay and interassay (*n* = 16) coefficient of variation (CV) is <10% for OPG. The intraassay (*n* = 16) CV is between 5–7% and interassay (*n* = 10) CV is 7–9% for RANK-L.

OPG was measured using a commercial sandwich-type ELISA to directly determine OPG in serum. In this assay, two highly specific antibodies against OPG are used. The binding antibody is attached to the microtiterplatemicrotiter wells, and the detection antibody is labeled with biotin. In a first incubation step, the samples and the biotinylated antibody against OPG react simultaneously with the pre-coated antibody on the microtiter plate. A sandwich-type complex is found consisting of the binding antibody on the plate, OPG and the biotinylated detection antibody. To remove all unspecific bound substances, a washing step is carried out. In a second step, streptavidin–peroxidase is added, reacting to the detection antibody. After another washing step, the solid phase is incubated with the substrate, TMB. An acidic stopping solution is subsequently added—the blue color changes to yellow. The intensity of the yellow color is directly proportional to the concentration of OPG in the sample. A dose-response curve of the absorbance units (at 450 nm) versus concentration is generated. OPG, present in the samples, was determined directly from this calibration curve (Immundiagnostik, Bensheim, Germany).

Bone formation markers, P1NP, were analyzed on a Roche Cobas e411 using enzyme-linked immunosorbent assay (ELISA). The within-day assay CV was 1.3 to 3.0% for a concentration range of 12.8–1140 ug/L (ng/mL), while the between-day assay CV was 12.2–4.1 % for a concentration range of 12.8–1140 ug/L (ng/mL). 

BMD of the second to the fourth lumbar spine (L2–L4), including gender- and age-corrected standard deviation score (z-score), was measured using dual-energy X-ray absorptiometry (DXA) with the use of the Hologic^®^ bone densitometer. Areal BMD of the second to the fourth lumbar spine (L2–L4), including gender- and age-corrected standard deviation score (z-score), was measured using dual-energy X-ray absorptiometry (Norland DEXA model XR-36; coefficient of variation 1%). Volumetric BMD, a function of bone mineral content per volume of bone, was calculated assuming that the spine is cylindrical (π × radius^2^ × height). The radius was derived from the width of the vertebra as measured by the dual-energy X-ray absorptiometry software [23].

### 2.3. Statistical Analysis

Descriptive statistics of age, gender, ethnicity, smoking status, the severity of depression, duration of MDD and SSRI use, body mass index (BMI), weekly exercise duration, levels of lipid, age of menarche and duration of menstrual cycle and menstruation were calculated. The distribution of data was assessed by the Shapiro–Wilk normality test. All data demonstrated normal distribution except menarche age, menstruation duration, P1NP, OPG, RANKL and RANKL/OPG ratio that demonstrated skewed distribution (*p* < 0.05 for medicated patients with MDD and healthy controls). BMD of the lumbar spine, left and right hips, mean levels of P1NP, RANKL. OPG, RANKL/OPG ratio were compared between female patients suffering from MDD and healthy controls using Student’s *t*-test for normal distribution or the Mann–Whitney U test for skewed data. Statistical significance was defined as a 2-tailed *p*-value < 0.05. All statistical analyses were performed using the SPSS program (Version 27.0, Chicago, IL, USA).

### 2.4. Ethics Approval

The study protocol was reviewed and approved by the Domain Specific Review Board (DSRB), which is the Institutional Review Board (IRB) of the National Healthcare Group, Singapore (DSBR reference: 2006/00464). Written informed consent was obtained from participants. The study was conducted according to the guidelines of the Declaration of Helsinki. Informed consent was obtained from all subjects involved in the study.

## 3. Results

### 3.1. Comparison of Characteristics between Patients and Healthy Controls

Table 1 compares the characteristics between medicated patients with MDD and healthy controls. We recruited 90 participants in total, including medicated patients with MDD and healthy controls. The study population was comprised of 66 Chinese (73.3%), 12 Malay (13.33%), 6 Indian (6.67%) and 6 participants from other ethnicities (6.67%). The ethnic distribution of our participants is representative of the ethnic distribution of Singaporeans. There were no significant differences in age (*p* = 0.757), ethnicity (*p* = 0.1494), smoking status (*p* = 0.17), BMI (*p* = 0.362), cholesterol (*p* = 0.85), triglycerides (*p* = 0.852), HDL-c (*p* =0.472), LDL-c (*p* =0.995), exercise duration (*p* = 0.975), menarche age (*p* = 0.876) and duration of menstruation (*p* = 0.376). Medicated patients with MDD had significantly higher HAM-D scores as compared with healthy controls (*p* < 0.001). 

### 3.2. Comparison of Bone Mineral Density between Medicated Patients with MDD and Healthy Controls

Table 2 compares the BMD between MDD patients receiving SSRIs and healthy controls. There were no significant differences in the mean BMD in the lumbar spine (*p* = 0.617), left hip (*p* = 0.181) and right hip (*p* = 0.784).

### 3.3. Comparison of Bone Formation and Metabolism Markers between Medicated Patients Suffering from MDD and Healthy Controls

Table 3 compares the bone markers between MDD patients receiving SSRIs and healthy controls. There were no significant differences in median P1NP (*p* = 0.635), OPG (*p* =0.545), RANKL (*p* = 0.279) and RANKL/OPG ratio (*p* = 0.279).

## 4. Discussion

Herein, our study found that premenopausal Singaporean women treated with SSRIs showed similar BMD at three skeletal sites (i.e., lumbar spine, left hip and right hip), levels of bone formation (i.e., P1NP) and bone metabolism (i.e., OPG, RANKL, RANKL/OPG ratio) markers when compared to healthy controls. Our findings on multi-ethnic Singaporean patients are in accordance with previous studies conducted in Americans that found no association between antidepressant use and bone loss in middle-aged women [6]. For Americans, the mean lumbar BMD for healthy controls and patients with MDD who received SSRI treatment were 1.05 and 1.07, respectively [6]. In our study involving Singaporeans, the mean lumbar BMD for healthy controls and patients with MDD who received SSRI treatment were 1.0 and 1.0, respectively. For Americans, the mean hip BMD for healthy controls and patients with MDD who received SSRI treatment were 0.94 and 0.99 respectively. For Japanese, the mean hip BMD for healthy controls and patients with MDD who received SSRI treatment were 0.95 and 0.95, respectively [24]. Our study shows similar results. The mean hip BMD for healthy controls and patients with MDD who received SSRI treatment were 0.8 and 0.9, respectively. Similarly, a study conducted on French women found no association between depression and anxiety or SSRI treatment [7] and another study conducted on Japanese women found antidepressant use was not associated with reduced BMD [24]. The above findings have clinical implications and call for caution in associating SSRI antidepressant use and osteoporosis, as this would reduce adherence to SSRI antidepressants, increasing the risk of relapse of MDD and suicide, and increasing the direct and indirect costs of managing MDD.

The methodological aspects of prior studies reporting an association between antidepressant use and osteoporosis need to be interpreted in light of methodological differences across studies. First, some of these studies mainly focused on elderly patients [25,26,27,28,29], and their findings should not be generalized to premenopausal women and adults. Second, some of the studies examined the database of patients who presented with hip fracture and identified the association between retrospective antidepressant use and hip fracture [30,31]. This association could be a spurious relationship due to either coincidence or the presence of confounding factors (for example, poor calcium intake and other comorbidities). Moreover, more than 80% of medicated patients in the current study with MDD and healthy controls were non-smokers because smoking is less common in Singaporean patients with depression than in Western countries [32]. The low consumption of tobacco avoids the confounding effect of nicotine on osteopenia [33].

There was no significant difference in the levels of bone formation and bone metabolism markers between medicated patients with MDD and healthy controls. P1NP is the most specific bone formation marker. A previous study found that fluoxetine and escitalopram showed mixed effects on the levels of P1NP [10]. Another study suggested that escitalopram did not significantly alter the P1NP levels and bone metabolism in the short term [34]. RANKL is a key factor for osteoclast activation and bone metabolism. A previous study found fluoxetine-induced RANKL overproduction in postmenopausal women [11], but our study did not find any difference in RANKL levels between premenopausal medicated women with MDD and healthy controls. OPG inhibits RANK–RANKL interactions, thus suppressing osteoclastogenesis and bone resorption [35], and there was no difference in OPG levels between medicated patients with MDD and healthy controls in this study. Furthermore, there was no difference in the RANKL/OPG ratio between the two groups. Our findings suggest that medicated patients with MDD and healthy controls had similar bone mass and skeletal integrity.

This study has several limitations. First, the sample size is relatively small compared to previous studies based on a retrospective database or community because this study was conducted in a university hospital or tertiary center. Second, this cross-sectional study could not establish the temporal relationship between SSRI use and change of BMD, and further research is required to monitor BMD and bone markers before and after initiation of SSRI treatment. Nevertheless, the mean duration of antidepressant use was 33 months, and the BMD of female patients on maintenance SSRI treatment and healthy controls was compared. This finding can be applied to patients with MDD who are on long-term SSRI treatment. Third, the levels of vitamin D and calcium in medicated patients with MDD and healthy controls were not quantified. Fourth, the findings of this study cannot be generalized to non-Singaporean populations, medicated patients with MDD who suffer from other comorbidities such as substance misuse and eating disorders such as anorexia nervosa, postmenopausal women and elderly men who suffer from MDD. Finally, this study only focused on SSRIs, and further research that compares different classes of antidepressants (for example, SNRI, TCA) with equal sample sizes is required, because the different mechanisms of action of antidepressants may have a different impact on BMD and bone markers.

## 5. Conclusions

This study compared the BMD between medicated women with MDD receiving SSRI treatment at a university hospital in Singapore and healthy controls from the community. There were no significant differences in the mean BMD between patients and controls in the lumbar spine, left hip and right hip. Furthermore, there were no differences in the bone formation marker (P1NP) and bone metabolism markers (RANKL, OPG and RANKL/OPG ratio) between medicated patients with MDD and controls. Chronic SSRI use might not be associated with low BMD and bone formation markers in Singaporean premenopausal women who suffered from MDD. This finding may help Singaporean patients with MDD to make an informed decision when considering the risks and benefits of SSRI treatment.

## Figures and Tables

**Table 1:** Characteristics of patients with MDD receiving SSRIs and healthy controls.

	Healthy Control (*n* = 45)	Major Depressive Disorder (*n* = 45)	*p*-Value
Age (years)	38.1 ± 9.2	37.6 ± 7	0.758
Ethnicity			0.149
Chinese	33 (73.3%)	33 (73.3%)	
Malay	6 (13.3%)	6 (13.3%)	
Indian	1 (2.2%)	5 (11.1%)	
Others	5 (11.1%)	1 (2.2%)	
Smoking status			0.170
Non-smoker	42 (93.3%)	36 (80%)	
Current smoker	1 (2.2%)	4 (8.9%)	
Past smoker	2 (4.4%)	5 (11.1%)	
Scores of Hamilton Depression Rating Scale (HAM-D) ^a^	1.7 ± 2.3	14.5 ± 7.1	**≤0.001**
Duration of MDD (months)		57.2 ± 59.9	
Duration of SSRI use (months)		33.2 ± 42.5	
Body Mass Index (BMI)	22.7 ± 3.8	23.6 ± 5	0.367
Cholesterol (mmol/L) ^a^	5.3 ± 1.1	5.2 ± 1	0.850
Triglycerides (mmol/L) ^a^	1.1 ± 0.9	1.1 ± 0.5	0.852
HDL-c (mmol/L) ^a^	1.5 ± 0.3	1.5 ± 0.4	0.472
LDL-c (mmol/L) ^a^	3.3 ± 0.9	3.2 ± 0.9	0.995
Exercise duration (hours/week) ^a^	1.4 ± 1.4	1.4 ± 3.2	0.975
Menarche age (years) ^a,b^	12.7 (11–15)	12 (9–21)	0.876
Menstruation duration (days) ^a,b^	5 (3–7)	5 (1.5–8)	0.376

*p*-values ≤ 0.05 are in bold. ^a^ Complete data was not obtained (HAM-D: Healthy control, *n* = 42; Major depressive disorder, *n* = 45. Cholesterol: Healthy control, *n* = 42; Major depressive disorder, *n* = 32. Triglycerides: Healthy control, *n* = 42; Major depressive disorder, *n* = 32. HDL-c: Healthy control, *n* = 42; Major depressive disorder, *n* = 32. LDL-c: Healthy control, *n* = 41; Major depressive disorder, *n* = 32. Exercise duration: Healthy control, *n* = 38; Major depressive disorder, *n* = 45. Menarche age: Healthy control, *n* = 38; Major depressive disorder, *n* = 47. Menarche age: Healthy control, *n* = 38; Major depressive disorder, *n* = 45. Menstruation duration: Healthy control, *n* = 38; Major depressive disorder, *n* = 43.) ^b^ For skewed data, the Mann–Whitney U test was performed.

**Table 2:** Comparison of the mean BMD between patients with MDD receiving SSRI treatment and healthy controls.

	Healthy Control (*n* = 45)	Major Depressive Disorder (*n* = 45)	*p*-Value
Lumbar BMD	1.041 ± 0.173	1.024 ± 0.145	0.617
Left hip BMD	0.823 ± 0.117	0.861 ± 0.146	0.181
Right hip BMD	0.843 ± 0.117	0.850 ± 0.135	0.784

**Table 3:** Comparison of the median bone formation and metabolism markers between patients with MDD receiving SSRI treatment and healthy controls.

	Healthy Control (*n* = 45)	Major Depressive Disorder (*n* = 45)	*p*-Value
P1NP	35.9 (5–105)	37.3 (5–116.5)	0.635
OPG	2.6 (0.1–8.6)	2.7 (1.4–7.4)	0.545
RANKL	23.4 (0.2–(13.5 × 10^4^))	2178.9 (0.2–(69.1 × 10^4^))	0.279
RANKL/ONG ratio	4.1 (0–(48.1 × 10^4^))	741.4 (0–(18.8 × 10^4^))	0.279

Complete data were not obtained (P1NP: Healthy Control, *n* = 41; Major Depressive Disorder, *n* = 44.ONG: Healthy Control, *n* = 38; Major Depressive Disorder, *n* = 43. RANK: Healthy Control, *n* = 36; Major Depressive Disorder, *n* = 41. RANKL/ONG ratio: Healthy Control, *n* = 33; Major Depressive Disorder, *n* = 42.).

## Data Availability

The data presented in this study are available on request from the corresponding author. The data are not publicly available due to ethical considerations.
